# Adjusted Donor Age: A Clinical Score to Support Organ Acceptance Decisions in Deceased-Donor Kidney Transplantation

**DOI:** 10.3389/ti.2024.13477

**Published:** 2024-11-07

**Authors:** Rupert Bright, Christoph F. Mahler, Anamika Adwaney, Dhriti Dosani, Emma Morganti, Felix Friedl, Christian Nusshag, Claudius Speer, Louise Benning, Daniel Göth, Matthias Schaier, Claudia Sommerer, Markus Mieth, Arianeb Mehrabi, Martin Zeier, Christian Morath, Frank J. M. F. Dor, Florian Kälble, Damien Ashby

**Affiliations:** ^1^ West London Renal and Transplant Centre, Imperial College Healthcare NHS Trust, London, United Kingdom; ^2^ Department of Nephrology, University Hospital Heidelberg, Heidelberg, Germany; ^3^ Department of General, Visceral and Transplantation Surgery, University Hospital Heidelberg, Heidelberg, Germany; ^4^ Department of Surgery and Cancer, Imperial College, London, United Kingdom

**Keywords:** kidney transplantation, deceased donor, patient empowerment, acceptance, decision making

## Abstract

As transplant programmes have evolved to allow a wider donor pool, organ acceptance decisions have become increasingly complex and lack transparency and equality. Clinical scoring tools exist but there is limited consensus on their use. From a prospective observation of consecutive deceased-donor kidney offers in a large urban transplant centre, a simple score was developed based on donor age and other risk characteristics, excluding ischemia time and graft histology. The score was validated in subsequent cohorts of consecutive offers in the United Kingdom and Germany. In the development cohort of 389 kidney offers, 110 (28%) were transplanted and 175 (45%) declined. Nine risk factors were incorporated into a score based on age, but adjusted for the number of risk factors present, making an “adjusted donor age,” with offers separated into equal quintiles by decade. The score was validated in a UK cohort of 380 subsequent offers, and a German cohort of 431 offers. In both cohorts adjusted donor age discriminated between favourable and poor post-transplant outcomes (C-statistic 0.77 in the United Kingdom, 95% CI 0.65–0.88, and 0.71 in Germany, 95% CI 0.64–0.77). Adjusted donor age is a simple score quantifying deceased donor kidney quality, which is consistent with current practice and predicts post-transplant outcome.

## Graphical Abstract |

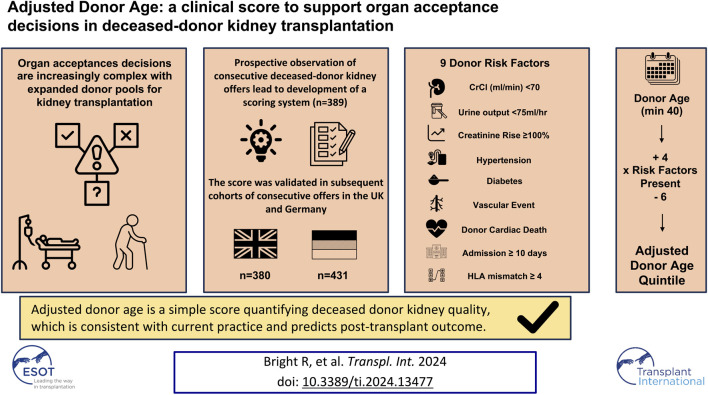

## Introduction

As transplant programmes evolve to tackle the rising burden of end stage kidney disease, the age and comorbidity of those considered as deceased donors has increased [1, 2]. Organ acceptance decisions are therefore increasingly complex, with practices varying significantly between clinicians and institutions, with limited patient involvement in the decision-making process, leading to a system that lacks transparency and equality [3]. It is a major challenge therefore for clinicians to ensure that the appropriate organ acceptance decisions are made, and that this process is accountable and communicated effectively with patients.

A number of studies have identified donor factors predictive of post-transplant outcome, including donor age and aspects of medical history such as hypertension, stroke, diabetes and kidney function [4]. To support acceptance decisions, clinical tools have been devised which combine several factors into a single numerical score, to indicate quality of the donor kidney, including the Deceased Donor Score [5], Donor Risk Grade [6], Kidney Donor Risk Index [7] and UK Kidney Donor Risk Index [8].

However, scoring systems so far developed have no meaning which is intuitive to patients, and are not calibrated to the donor pool, with over half of all offers placed in the highest category of risk. They provide limited assistance therefore in comparing the current offer to possible future offers, and do little to enhance patient understanding or facilitate a shared decision. There is limited consensus therefore on the use of such tools in the organ acceptance decision process [9]. The hypothesis of this study is that it is possible to develop a valid clinical tool based on age, which can easily be understood by patients, to assist with shared decision making in organ acceptance from deceased kidney donors.

## Patients and Methods

In two large urban transplant centres, three sequential studies were carried out. The first was a prospective observation of deceased-donor kidney offers in a single UK centre during 2018, collecting donor and recipient characteristics, decisions and clinical outcomes. A simple score was developed based on donor age and other offer characteristics, termed “adjusted donor age,” and calibrated to separate all offers into quintiles according to quality. The second was a validation of this score in a subsequent UK cohort of consecutive offers. The third was a further validation in a cohort of consecutive offers in Germany. As a retrospective study of routinely available data, the protocols were approved by the National Research Ethics Service (IRAS Ref 308076) in the United Kingdom, and the local Ethics Committee in Germany (S-187/2022) without requirements for individual consent.

Variables were selected from published donor-scoring literature (age, gender, comorbidities, donor cardiac death, cause of death, length of admission, HLA mismatch) supplemented with additional variables that are commonly considered (smoking, alcohol excess, proteinuria, cardiac arrest duration). Efforts were made to distinguish between acute kidney injury (using urine output and creatinine rise from baseline) and baseline creatinine clearance, which was estimated by the Cockcroft-Gault equation, using a simplified formula for adjusted body weight: (weight+70)/2 (male), (weight+55)/2 (female), and using the average of pre-admission, initial-admission and lowest-during-admission for baseline creatinine. Variables were subsequently excluded from consideration if there was limited or conflicting evidence for outcome prediction in prior literature and the development cohort. Ischaemia times were not considered since they are usually unknown at the time of the initial decision, and similarly graft histology was not included since it is so rarely available in either institution.

The unit of analysis was the offer of a donor kidney for a particular (named) recipient. Where both kidneys were offered from the same donor (for different recipients), they were considered as separate offers. In a minority of cases no recipient was specified by the allocation system and clinical teams were able to select any suitable recipient. Kidneys declined for one recipient but also thought unsuitable for any recipient were analysed as a single offer for that recipient. Initial acceptance decisions were made by clinicians only, via joint agreement between a nephrologist and a transplant surgeon. Decline decision types were defined as “exclusion” if due to a single qualitative factor occasionally with recipient involvement in the decision (e.g., recent donor cancer) or as “quality” if due to the combination of quantitative variables such as age, creatinine clearance and comorbidities, usually without involvement of the recipient. Decisions were defined as “other” if initially accepted but then not transplanted due to factors outside the control of the clinical team. Most commonly this would be due to a prolonged agonal phase in donation after circulatory death (DCD) donors, leading to withdrawal of the offer, but sometimes recipient factors were involved, for example, if the recipient was unwell or unavailable.

The influence of variables on acceptance decisions was assessed with Fisher’s exact test comparing transplanted with declined offers. Outcome prediction was assessed by logistic regression with poor outcome defined as organ failure or GFR below 30 mL/min/1.72 m^2^ at 3 months after transplantation, using an average of three consecutive outpatient creatinine measurements. Analyses were performed using Microsoft Excel, JASP (Jeffreys’ Amazing Statistics Program, JASP Team, 2020) and RStudio (R Team, 2021).

## Results

The development cohort consisted of 389 consecutive kidney offers, from 302 deceased donors (aged 6–84). The majority of offers (93%) were for specified recipients (aged 24–78), with donor and recipient characteristics for all offers provided in Table 1. Out of all offers, 110 (28%) were transplanted, 175 (45%) were declined and 104 (27%) initially accepted but then not transplanted due to factors outside the control of the clinical team. Of the 175 offers declined by the clinical team, 43 (11% of all offers) were declined due to an exclusion factor, with the remaining 132 (34% of all offers) declined due to quality concerns.

**TABLE 1:** Offer characteristics, decision and outcome post-transplant in the development cohort (N = 389).

	Acceptance decisions (N = 389)				Post-transplant outcome univariate (N = 110)			Selection^e^
	All offers	Transplant (110)	Declined (175)	*p*-value^a^	OR^b^	95% CI	*p*-value	
Donor								
Age (years)	60 (51–71)	56 (47–67)	68 (59–76)	0.001	1.07	1.03–1.12	0.002	
Male gender	205 (53)	59 (54)	84 (48)	0.330	0.40	0.18–0.91	0.066	Excluded
CrC (mL/min) continuous^c^	86 (70–112)	89 (74–113)	75 (60–92)	0.002	0.96	0.94–0.98	0.000	
<70^d^	102 (26)	24 (22)	67 (38)	0.006	6.61	2.75–15.9	0.000	
Creatinine ≥100% rise	42 (11)	8 (7)	29 (17)	0.029	0.54	0.09–3.31	0.579	
Urine <75 mL/h	143 (37)	36 (33)	77 (44)	0.082	4.02	1.77–9.11	0.005	
Proteinuria (>1+)	189 (49)	41 (37)	113 (65)	0.000	1.11	0.43–2.84	0.836	Excluded
Hypertension	168 (43)	34 (31)	94 (54)	0.000	2.78	1.24–6.24	0.037	
Diabetes	53 (14)	8 (7)	31 (18)	0.014	1.35	0.33–5.50	0.725	
Vascular event	42 (11)	2 (2)	31 (18)	0.000	2.02	0.26–15.8	0.572	
Alcohol excess	69 (18)	18 (16)	31 (18)	0.873	0.40	0.12–1.31	0.204	Excluded
Smoking	158 (41)	40 (36)	68 (39)	0.802	0.77	0.32–1.88	0.631	Excluded
Donor cardiac death	151 (39)	35 (32)	68 (39)	0.310	3.34	1.49–7.51	0.014	
Stroke (cause of death)	167 (43)	46 (42)	78 (45)	0.806	0.74	0.33–1.66	0.536	Excluded
Arrest duration >30 min	67 (17)	21 (19)	32 (18)	0.876	0.99	0.97–1.01	0.444	Excluded
Admission ≥10 days	41 (11)	6 (6)	21 (12)	0.095	4.42	1.08–18.0	0.082	
HLA mismatch ≥4	82 (21)	24 (22)	37 (21)	0.882	1.55	1.01–2.37	0.093	
Recipient (N = 363)								
Age (years)	56 (48–63)	55 (47–65)	56 (47–62)	0.535	1.03	0.99–1.08	0.114	
Weight (kg)	75 (66–85)	74 (64–87)	75 (67–86)	0.639	1.05	1.02–1.09	0.003	
CRF > 50%	80 (22)	23 (20.9)	33 (22)	0.647	0.75	0.23–2.48	0.636	
Wait time (years)	3.4 (2.1–5.0)	2.8 (1.7–4.2)	3.5 (2.2–4.9)	0.009	1.16	0.93–1.45	0.193	

Data provided as number (%) or median (IQR), results shaded if *p* < 0.10. HLA, human leukocyte antigen; CRF, calculated reaction frequency.

^a^ *p* value comparing transplanted with declined.

^b^ OR, odds ratio for poor outcome (GFR <30) at 3 months.

^c^ CrC, creatinine clearance, as continuous variable.

^d^ CrC: creatinine clearance, as threshold dependent on recipient weight: 60 (<65 kg), 70 (65–85 kg), 80 (>85 kg).

^e^ Variables excluded as contributors to adjusted donor age are shown (see text).

Several donor characteristics were associated with acceptance decisions including age, creatinine clearance, creatinine rise, urine output, proteinuria, hypertension, diabetes, vascular events and length of the donor’s hospital admission (Table 1). Decisions had to be made within a short time after receiving the offer, with 39% of decisions made between 21:00 and 06:00.

After 3 months, of the 110 recipients transplanted, 87 (79%) had a favourable clinical outcome, whereas 17 had poor transplant function (GFR below 30 mL/min/1.72 m^2^) and 6 kidneys had permanently failed. Donor characteristics predictive of poor transplant outcome (GFR <30 or failure) included age, gender, creatinine clearance, urine output, hypertension, cardiac death, length of admission and HLA mismatch (Table 1). Greater recipient weight also predicted poor outcome. Some of the counterintuitive relationships between donor factors and transplant outcome may be explained by collinearity between factors (Supplementary Table S1). In a sensitivity analysis, similar prediction characteristics were found using outcome at 12 months post-transplantation (Supplementary Table S2).

**FIGURE 1:**
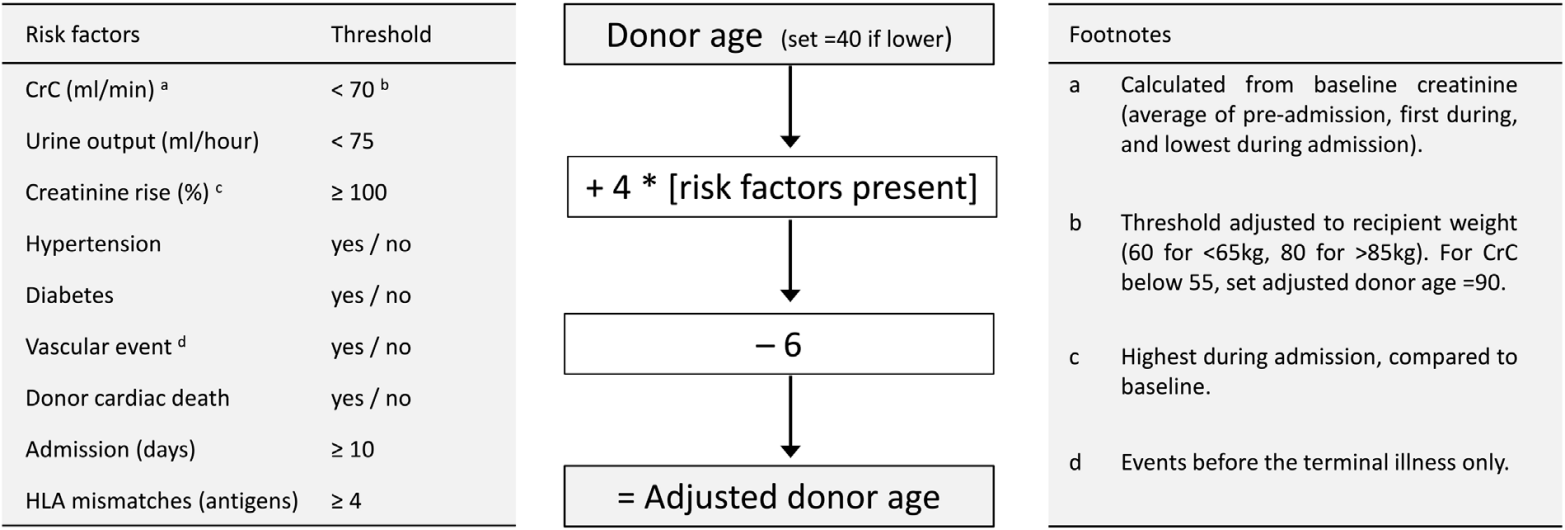
Calculation of adjusted donor age. Left panel describes 9 risk factors with thresholds for their presence. Central panel describes calculation of adjusted donor age. Right panel contains clarifying footnotes. CrC, creatinine clearance (by Cockroft-Gault formula); HLA, human leucocyte antigen.

Donor variables without predictive ability, which were therefore excluded from further analysis, included proteinuria, alcohol excess, smoking, death from stroke and cardiac arrest duration. Gender was also excluded since its effect disappeared after adjustment for creatinine clearance (calculation of which includes gender). The number of kidneys transplanted from donors with diabetes or prior vascular events was small – these factors, which are increasingly prevalent amongst deceased donors, were retained since they exerted a marked influence on acceptance decisions, and would therefore be under-estimated as predictors of post-transplant outcome. Nine risk factors were therefore incorporated into a score based on age, but adjusted for the number of risk factors present, making an “adjusted donor age” (Figure 1). Thresholds and coefficients were selected to optimise prediction, with risk factors scaled by 4 years, so that the score remains largely age-dependent (like other scores such as KDRI) with 6 years subtracted to centre the distribution. Since recipient weight was a strong predictor of outcome, weight-dependent thresholds were used for donor creatinine clearance.

As expected, the adjusted donor age was still strongly correlated with donor age with coefficient of determination (*R* ^2^) 0.75, suggesting that 75% of the variation in adjusted donor age was explained by donor age. Age was therefore the dominant determinant of the score, as it is with published scoring systems: calculating the Kidney Donor Risk Index [7] and UK Kidney Donor Risk Index [8] in this cohort, gave coefficients of determination of 0.76 and 0.72, respectively for the relationship with donor age. Using published risk thresholds for either score however, over half of all offers from this cohort fell into the highest risk category, whereas offers were separated approximately into quintiles using decade of adjusted donor age (Table 2).

**TABLE 2:** Offer characteristics by adjusted donor age and established risk scores in the development cohort (N = 389).

	Risk category				
	1	2	3	4	5
Adjusted donor age	<49	50–59	60–69	70–79	>80
Offers (number)	89 (23)	81 (21)	62 (16)	79 (20)	78 (20)
Age (years)	42 (32–49)	54 (51–57)	61 (59–65)	71 (68–74)	75 (69–78)
Risk factors present^a^	1 (0–2)	2 (1–2.5)	2 (2–3)	3 (2–3)	4 (3–5)
US KDRI^b^	<0.75	0.75–0.91	0.91–1.11	1.11–1.39	>1.39
Offers (number)	28 (7)	25 (6)	51 (13)	67 (17)	218 (56)
Age (years)	28 (20.5–32.5)	39 (35–46)	50 (47–53.5)	54 (51.5–58)	70 (64–75)
Risk factors present^a^	1.5 (0–3)	1 (0–2)	1 (0–3)	2 (1–2.5)	3 (2–5)
UK KDRI^c^	<0.87	0.87–1.02	1.02–1.34	>1.34	
Offers (number)	41 (11)	56 (14)	85 (22)	207 (53)	
Age (years)	28 (23–35)	53.5 (50–56)	52 (47–55)	70 (65–76)	
Risk factors present^a^	1 (0–2)	2 (0–2)	2 (1–3)	3 (2–5)	

Data provided as N (%) or median (IQR). KDRI, kidney donor risk index.

^a^ Risk factors are those given in Figure 1.

^b^ Rao, Transplantation, 2009, scaled to median offer and using updated quintile boundaries from 2018 USA cohort.

^c^ Watson, Transplantation, 2012, using original quartile boundaries from 2000 to 2007 UK cohort.

The adjusted donor age score was validated in separate cohorts from the UK and Germany. The UK validation cohort consisted of 377 consecutive offers, of which 96 (25%) were transplanted, 176 (47%) declined and 105 (28%) initially accepted but then not transplanted. Three months after transplantation, outcomes in this cohort were similar, with a favourable 3-month clinical outcome (GFR above 30 mL/min/1.72 m^2^) was seen in 78 recipients (81%). All risk factors were validated in this cohort by association with acceptance decisions or outcome or both, and greater recipient weight remained marginally predictive of poor outcome (Table 3).

**TABLE 3:** Offer characteristics, decision and outcome post-transplant in the UK validation cohort.

	Acceptance decisions (N = 377)				Post-transplant outcome univariate (N = 96)			Post-transplant outcome multivariate^a^		
	All offers	Transplant (96)	Declined (176)	*p*-value^b^	OR^c^	95% CI	*p*-value	OR^c^	95% CI	*p*-value
Donor										
Age (years)	61 (51–71)	55 (48–66)	66 (54–72)	0.000	1.06	1.01–1.11	0.016	1.09	1.03–1.16	0.006
CrC (mL/min) < 70^d^	92 (24)	21 (22)	58 (33)	0.069	4.00	1.33–12.1	0.014			
Creatinine ≥100% rise	46 (12)	8 (8)	34 (19)	0.022	0.60	0.07–5.18	0.640			
Urine <75 mL/h	183 (49)	36 (38)	99 (56)	0.004	1.08	0.38–3.08	0.893			
Hypertension	153 (41)	30 (31)	92 (52)	0.001	1.10	0.37–3.29	0.859			
Diabetes	42 (11)	5 (5)	34 (19)	0.001	1.00	0.00–>100	0.993			
Vascular event	42 (11)	3 (3)	30 (17)	0.000	1.00	0.00–>100	0.991			
Donor cardiac death	189 (50)	34 (35)	94 (53)	0.005	2.81	0.99–8.01	0.053			
Admission ≥10 days	31 (8)	3 (3)	16 (9)	0.082	9.50	0.81–>100	0.073			
HLA mismatch ≥4	156 (41)	49 (51)	61 (35)	0.010	6.32	1.33–30.1	0.021			
Risk factors present^e^					1.67	1.08–2.58	0.020	1.72	1.02–2.89	0.042
Recipient (N = 333)										
Weight (kg)	75 (65–88)	78 (65–89)	75 (66–90)	0.729	1.02	1.00–1.04	0.095	1.04	1.01–1.07	0.006

Data provided as number (%) or median [IQR], results shaded if *p* < 0.10. HLA, human leukocyte antigen.

^a^ multivariable model adjusted for variables shown.

^b^ *p* value comparing transplanted with declined.

^c^ OR, odds ratio for poor outcome (GFR<30) at 3 months.

^d^ CrC, creatinine clearance, as threshold dependent on recipient weight: 60 (<65 kg), 70 (65–85 kg), 80 (>85 kg).

^e^ Total number of donor risk factors (from the above list) present.

The German validation cohort included 431 consecutive offers, of which 173 (40%) were transplanted, 224 (52%) declined and 34 (8%) initially accepted but then not transplanted (a smaller category due to the absence of DCD donors). A favourable 3-month clinical outcome (GFR above 30 mL/min/1.72 m^2^) was seen in 146 (84%). Apart from vascular events and length of admission, risk factors were also validated in this cohort by association with acceptance decisions or outcome or both (Table 4).

**TABLE 4:** Offer characteristics, decision and outcome post-transplant in the German validation cohort.

	Acceptance decisions (N = 431)				Post-transplant outcome univariate (N = 173)			Post-transplant outcome multivariate^a^		
	All offers	Transplant (173)	Declined (224)	*p*-value^b^	OR^c^	95% CI	*p*-value	OR^c^	95% CI	*p*-value
Donor										
Donor age (years)	63 [52–78]	60 [50–72]	68 [56–80]	0.000	1.08	1.04–1.13	0.000	1.07	1.03–1.11	0.001
CrC (mL/min) < 70^d^	123 (29)	66 (32)	57 (25)	0.170	2.12	0.91–4.92	0.080			
Creatinine ≥100% rise	75 (17)	23 (11)	52 (23)	0.001	0.34	0.04–2.65	0.301			
Urine <75 mL/h	109 (25)	39 (19)	70 (31)	0.004	1.87	0.71–4.93	0.205			
Hypertension	225 (52)	104 (50)	121 (54)	0.492	6.24	2.06–18.9	0.001			
Diabetes	62 (14)	21 (10)	41 (18)	0.023	4.29	1.49–12.4	0.007			
Vascular event	53 (12)	23 (11)	30 (13)	0.566	1.09	0.29–4.06	0.896			
Admission ≥10 days	37 (9)	15 (7)	22 (10)	0.435	0.43	0.05–3.45	0.426			
HLA mismatch ≥4	92 (21)	53 (26)	39 (17)	0.050	4.61	1.96–10.9	0.000			
Risk factors present^e^					1.97	1.40–2.78	0.000	1.50	1.01–2.22	0.045
Recipient (N = 305)										
Weight (kg)	75 [64–88]	76 [65–88]	74 [63–87]	0.728	1.00	0.97–1.02	0.906			

Data provided as number (%) or median [IQR], results shaded if *p* < 0.10. HLA, human leukocyte antigen.

^a^ multivariable model adjusted for variables shown.

^b^ *p* value comparing transplanted with declined.

^c^ OR, odds ratio for poor outcome (GFR<30) at 3 months.

^d^ CrC, creatinine clearance, as threshold dependent on recipient weight: 60 mL/min (<65 kg), 70 mL/min (65–85 kg), 80 mL/min (>85 kg).

^e^ Total number of donor risk factors (from the above list) present.

**FIGURE 2:**
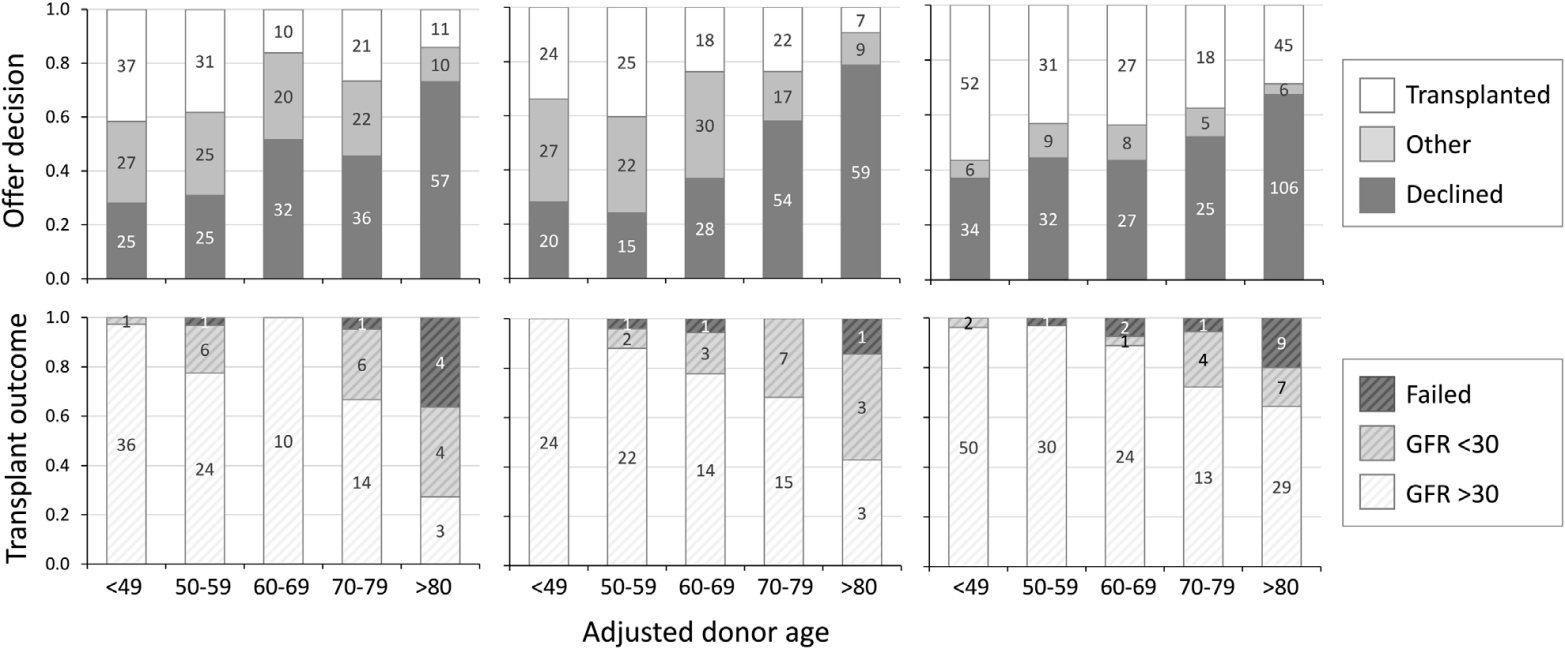
Offer decision and transplant outcome by adjusted donor age. Offer decision (upper panels) and transplant outcome at 3 months (lower panels) by adjusted donor age, in UK development cohort (left), UK validation cohort (centre) and German validation cohort (right). Decision “other” = offers accepted but not transplanted due to non-donor factors. GFR: glomerular filtration rate (ml/min/1.72 m^2^).

The ability of adjusted donor age to predict both acceptance decision and outcome after transplantation was analysed by calibration and discrimination in both validation cohorts. In the UK validation cohort adjusted donor age was well calibrated to decisions with each quintile increasing the rate of decline (28%, 24%, 37%, 58% and 79%) with OR 1.91 per quintile (95% CI 1.62–2.27, Figure 2). In Germany the rate of decline similarly increased with each quintile of adjusted donor age (37%, 44%, 44%, 52% and 68%) with OR 1.36 per quintile (95% CI 1.20–1.54, Figure 2). In both cohorts adjusted donor age discriminated between decisions (C-statistic 0.74 in the UK, 95% CI 0.69–0.79, and 0.72 in Germany, 95% CI 0.68–0.76).

**FIGURE 3:**
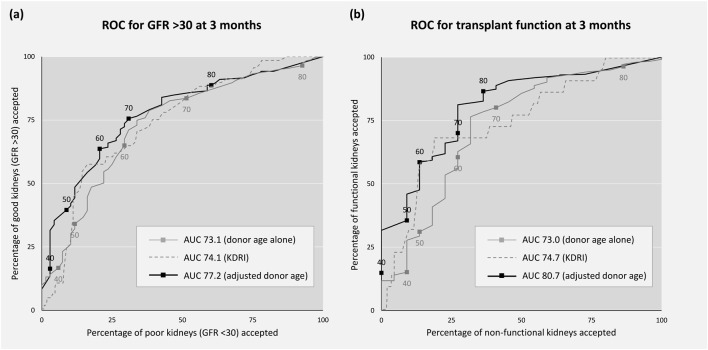
Receiver operating characteristic (ROC) curves. Sensitivity (good kidneys accepted) and 1-specificity (poor kidneys accepted) by threshold of donor age (grey), KDRI (grey dash) and adjusted donor age (black) in the combined cohort. Good outcome is defined as: **(A)** transplant GFR >30 at 3 months vs <30, and **(B)** transplant functioning at 3 months vs non-function. GFR, glomerular filtration rate (ml/min/1.72 m^2^), KDRI, kidney donor risk index.

In both cohorts adjusted donor age was calibrated to 3-month post-transplant outcome with each quintile increasing the likelihood of a poor outcome post-transplantation: 0%, 12%, 22%, 32% and 57% in the UK with OR 2.29 per quintile (95% CI 1.39–3.77, Figure 2) and 4%, 3%, 11%, 28% and 36% in Germany with OR 2.09 (95% CI 1.49–2.92, Figure 2). In both cohorts adjusted donor age discriminated between post-transplant outcomes (C-statistic 0.77 in the UK, 95% CI 0.65–0.88, and 0.71 in Germany, 95% CI 0.64–0.77). Receiver operating characteristic curves illustrating the ability of adjusted donor age to discriminate between favourable and poor outcome offers in the combined cohort is shown in Figure 3.

## Discussion

This paper describes a novel clinical tool for scoring the quality of a donor kidney offer, which is simple to calculate, calibrated to current acceptance practice, and predictive of outcome after transplantation. Such tools have appeared increasingly necessary as transplant procurement practices have evolved, from their conservative beginnings to an era of expanded criteria, allowing a much wider pool of potential donors. Studies have confirmed the survival benefit of transplantation from higher risk donors in selected recipients [10–12] but the greater variation in donor quality has made acceptance decisions increasingly complex and recipient-specific [13].

In making decisions about transplant offers, clinicians face a discrete choice within a skewed outcome distribution: kidney transplantation is usually successful, quickly and dramatically improving both quantity and quality of life. But an unsuccessful transplant, though much less common, may be fatal or disabling, or at best provide only a short reprieve from the burden of dialysis, often leaving the patient sensitised with limited prospects for re-transplantation. When to grasp opportunity, and when to play safe, is difficult to determine, not helped by the large number of donor and recipient factors which must be considered, the limited evidence base which lacks clear consensus, and the short timeframe within which decisions must be made, frequently at night.

Unsurprisingly marked variation in practice is seen between centres, with many kidneys being sequentially declined before finally being accepted and successfully transplanted – a process which leads to inequality, with marked regional differences in wait-time to transplantation [14]. That clinicians struggle with these decisions is also highlighted by the increased decline rate observed with night-time or weekend decisions [15, 16]. When clinical decisions are difficult due to complexity and time-pressure, yet stereotyped since the same concepts apply to every decision, a numerical tool offers a way to support decision making by framing the information, leading to consistency with less unwarranted variation.

Several clinical tools have previously been published which provide a numerical measure of the quality of a deceased donor kidney offer [5–8]. These have been developed from multivariate analysis of large registries, assessing the ability of donor characteristics to predict post-transplant outcome (either GFR at 6 months or time to transplant failure), leading to a score based heavily on donor age, but also including a small number of other characteristics, which partially overlap between studies. The “adjusted donor age” described in this study is similar to these tools in having age as the dominant contributor, and in predicting post-transplant outcome, but there are a number of important differences which are advantageous.

Firstly, currently available tools have boundaries which classify offers with respect to specific outcomes, rather than against the offer pool. Indeed, by the two most recently published tools [7, 8], over half of the offers in the development cohort would fall into the highest risk category. This is another significant limitation, since the acceptance decision is largely a comparative one, involving the likelihood of receiving a higher quality kidney offer within a short time. Decade boundaries of the adjusted donor age separate offers into quintiles, and although this might require recalibration over time or in different transplant programmes, the concept allows the current offer to be considered against future ones. The need for greater comparative thought in the decision process is obvious when one considers the significant number of offers declined despite belonging to the most favourable quintile, without an exclusion factor (i.e., on grounds of quality). Most often this arises from a failure to appreciate that the presence of several risk factors may be entirely offset by favourable donor age.

Secondly, although kidney function is an accepted predictor of post-transplant outcome, current tools base their estimate of function on terminal creatinine, thus failing to distinguish between chronic and acute kidney dysfunction, with recent studies suggesting the latter has only a much smaller impact on outcome. In contrast, the adjusted donor age is based on creatinine clearance estimated from baseline function, with creatinine rise and urine output as measures of acute dysfunction. Interestingly, whilst baseline creatinine clearance was a strong predictor of both decision and outcome in both cohorts, creatinine rise predicted decisions but not post-transplant outcome, suggesting it may be over-valued by decision makers. As the pool of potential donors expands, the frequency of offers with acute kidney injury will increase, so it will be increasingly important to distinguish between the large effect of chronic kidney disease and the lesser effect of acute kidney injury. Using different creatinine clearance thresholds according to recipient weight is also helpful in accounting for the negative impact of recipient size on outcome.

Finally, and most importantly, the adjusted donor age has an intuitive meaning: the age of the typical donor (with no unusual risk factors) equivalent in quality to the current offer. Since it can be readily understood, this would facilitate a discussion with the potential recipient and their involvement in the process. Though the value of shared decisions is widely appreciated, in current practice decisions are often made by clinicians only, or with limited patient involvement, in part due to the difficulty in expressing the balance of risk. Such communication is regarded as an essential part of the informed consent process [17] and linking new information to a familiar principal is believed to aid understanding [18]. This intuitive meaning, which also frames the offer within the whole distribution, may therefore facilitate patient understanding and involvement in a shared acceptance decision. Further study would be needed to assess patient feedback on the use of the score, as well as the influence on practice within centres.

There are several important limitations to this study, in particular the dual-centre and relatively small size for this type of study may reduce the ability to assess risk factors reliably. This is partially offset by the advantages of greater data granularity, the ability to understand the decision-making process, and consistency of other aspects of care which may influence outcome. There are clinical practice differences between the two centres, most notably in DCD transplantation which is not performed in Germany, leading, for example, to a much smaller group of accepted offers which did not proceed. Although the tool still validated reasonably well in the German cohort, it is possible that specific optimisation may enhance its utility in this setting.

The adjusted donor age score model does not incorporate graft histology or ischemia time. This is due to graft histology rarely playing a role in organ acceptance decisions in both the UK and Germany. Obtaining optimal pre-implantation graft histology results in the deceased donor setting is also challenging. Likewise, both warm and cold ischemia time are typically not known at the point of the initial offer acceptance decision, when the score is designed to be used. In the UK, a long warm ischaemia time is rare: typically, if there is an excessive delay between withdrawing life-support treatment and circulatory death, the retrieval is cancelled and the offer withdrawn.

As with all such tools, the outcome is only known for those kidneys which are accepted for transplantation, and a characteristic which is highly predictive of a declined offer, will be largely absent from the dataset of transplanted kidneys, and therefore have limited ability to also predict post-transplant outcome. The use of 3-month GFR as the outcome measure overlooks overall transplant survival, which is more meaningful to patients, though it may more easily capture the effect of donor-specific factors, the influence of which becomes diluted over time.

The study is specific to the UK and German deceased-donor transplant programs, and applicability beyond Europe is unknown. Whilst recalibration is likely to be necessary, the concept of an age-adjusted score to assist acceptance decisions and patient involvement should still be widely applicable. One drawback of all clinical tools including this study is outcome evidence restricted to those offers which proceeded to transplantation: the comparator needed is outcomes after not accepting the offer, such as time to the next offer, quality of that offer, and mortality or removal from the waiting list before transplantation is achieved. This outcome has received little research attention, but future studies will hopefully address this important knowledge gap.

The adjusted donor age score provides a transparent method of quantifying deceased donor kidney quality, which is consistent with current practice and predicts post-transplant outcome. Its intuitive meaning, which frames the offer against the donor distribution, may support organ acceptance decision making and facilitate meaningful patient involvement in the process.

## Data Availability

The data underlying this article cannot be shared publicly due to the privacy of individuals that were included in the study. The data will be shared on reasonable request to the corresponding author.

## Abbreviations

CRF, calculated rejection frequency; DCD, donation after circulatory death; GFR, glomerular filtration rate; HLA, anti-human leukocyte antigen; UK, United Kingdom.
