# Supplementary material for: Adjusted Donor Age: A Clinical Score to Support Organ Acceptance Decisions in Deceased-Donor Kidney Transplantation
Source: Transpl Int. 2024 Nov 7;37:13477. doi: 10.3389/ti.2024.13477 (PMC11578704; doi:10.3389/ti.2024.13477)
Supplement: Supplementary file 1 [file Table1.DOCX]

Supplementary Table S1. Associations between donor characteristics in the development cohort (N=389).

| Characteristic*: | Age>60 | Male | GFR | Creat | Urine | Prot | Hypert | Diabet | Vascul | Alcohol | Smoker | DCD | Stroke | Arrest | Admit | HLA |
| --- | --- | --- | --- | --- | --- | --- | --- | --- | --- | --- | --- | --- | --- | --- | --- | --- |
| Overall frequency: (N, %) | 204  52.4 | 205  52.7 | 103  26.5 | 42  10.8 | 143  36.8 | 75  19.3 | 168  43.2 | 53  13.6 | 42  10.8 | 69  17.7 | 158  40.6 | 151  38.8 | 167  42.9 | 67  17.2 | 41  10.5 | 82  21.1 |
| Frequency in subgroup below: (N, %, p value) | | |  |  |  |  |  |  |  |  |  |  |  |  |  |  |
| Age > 60 years |  | 98  48.0  .053 | 79  38.7  .000 | 16  7.8  .049 | 79  38.7  .399 | 49  24.0  .013 | 114  55.9  .000 | 36  17.6  .015 | 32  15.7  .001 | 17  8.3  .000 | 71  34.8  .014 | 108  52.9  .000 | 104  51.0  .000 | 27  13.2  .029 | 22  10.8  .869 | 47  23.0  .320 |
| Male gender | 98  47.8  .053 |  | 33  16.1  .000 | 21  10.2  .711 | 63  30.7  .009 | 36  17.6  .364 | 85  41.5  .469 | 31  15.1  .364 | 35  17.1  .000 | 50  24.4  .000 | 99  48.3  .001 | 84  41.0  .357 | 87  42.4  .836 | 38  18.5  .469 | 21  10.2  .841 | 51  24.9  .053 |
| GFR < 70 ml/min | 79  76.7  .000 | 33  32.0  .000 |  | 15  14.6  .151 | 45  43.7  .089 | 23  22.3  .360 | 53  51.5  .048 | 22  21.4  .008 | 14  13.6  .286 | 7  6.8  .000 | 34  33.0  .067 | 40  38.8  .997 | 44  42.7  .960 | 18  17.5  .937 | 10  9.7  .749 | 25  24.2  .354 |
| Creatinine > 100 % rise | 16  38.1  .049 | 21  50.0  .711 | 15  35.7  .151 |  | 23  54.8  .010 | 13  31.0  .042 | 17  40.5  .707 | 4  9.5  .412 | 2  4.8  .182 | 7  16.7  .847 | 10  23.8  .019 | 6  14.3  .000 | 9  21.4  .003 | 15  35.7  .000 | 4  9.5  .820 | 9  21.4  .953 |
| Urine < 75 ml/h | 79  55.2  .399 | 63  44.1  .009 | 45  31.5  .089 | 23  16.1  .010 |  | 35  24.5  .048 | 60  42.0  .709 | 23  16.1  .281 | 11  7.7  .132 | 28  19.6  .468 | 57  39.9  .817 | 78  54.5  .000 | 51  35.7  .027 | 18  17.5  .937 | 20  14.0  .091 | 30  21.0  .970 |
| Proteinuria > 1+ | 49  65.3  .013 | 36  48.0  .364 | 23  30.7  .360 | 13  17.3  .042 | 35  46.7  .048 |  | 37  49.3  .232 | 8  10.7  .406 | 7  9.3  .649 | 14  18.7  .815 | 38  50.7  .049 | 32  42.7  .446 | 34  45.3  .640 | 16  21.3  .294 | 9  12.0  .647 | 16  21.3  .952 |
| Hypertension | 114  67.9  .000 | 85  50.6  .469 | 53  31.5  .048 | 17  10.1  .707 | 60  35.7  .709 | 37  22.0  .232 |  | 31  18.5  .016 | 30  17.9  .000 | 19  11.3  .004 | 59  35.1  .054 | 71  42.3  .224 | 85  50.6  .008 | 16  9.5  .000 | 27  16.1  .002 | 43  25.6  .057 |
| Diabetes | 36  67.9  .015 | 31  58.5  .364 | 22  41.5  .008 | 4  7.5  .412 | 23  43.4  .281 | 8  15.1  .406 | 31  58.5  .016 |  | 14  26.4  .000 | 7  13.2  .353 | 23  43.4  .658 | 26  49.1  .100 | 18  34.0  .156 | 13  24.5  .130 | 7  13.2  .496 | 10  18.9  .671 |
| Vascular event (previous) | 32  76.2  .001 | 35  83.3  .000 | 14  33.3  .286 | 2  4.8  .182 | 11  26.2  .132 | 7  16.7  .649 | 30  71.4  .000 | 14  33.3  .000 |  | 8  19.0  .814 | 17  40.5  .984 | 18  42.9  .569 | 23  54.8  .101 | 6  14.3  .593 | 7  16.7  .171 | 9  21.4  .953 |
| Alcohol excess | 17  24.6  .000 | 50  72.5  .000 | 7  10.1  .000 | 7  10.1  .847 | 28  40.6  .468 | 14  20.3  .815 | 19  27.5  .004 | 7  10.1  .353 | 8  11.6  .814 |  | 46  66.7  .000 | 20  29.0  .065 | 23  33.3  .076 | 10  14.5  .508 | 7  10.1  .906 | 16  23.2  .636 |
| Smoker | 71  44.9  .014 | 99  62.7  .001 | 34  21.5  .067 | 10  6.3  .019 | 57  36.1  .817 | 38  24.1  .049 | 59  37.3  .054 | 23  14.6  .658 | 17  10.8  .984 | 46  29.1  .000 |  | 71  44.9  .041 | 67  42.4  .863 | 32  20.3  .191 | 26  16.5  .002 | 33  20.9  .938 |
| Donor Cardiac Death | 108  71.5  .000 | 84  55.6  .357 | 40  26.5  .997 | 6  4.0  .000 | 78  51.7  .000 | 32  21.2  .446 | 71  47.0  .224 | 26  17.2  .100 | 18  11.9  .569 | 20  13.2  .065 | 71  47.0  .041 |  | 51  33.8  .004 | 25  16.6  .781 | 25  16.6  .002 | 38  25.2  .116 |
| Stroke (cause of death) | 104  62.3  .000 | 87  52.1  .836 | 44  26.3  .960 | 9  5.4  .003 | 51  30.5  .027 | 34  20.4  .640 | 85  50.9  .008 | 18  10.8  .156 | 23  13.8  .101 | 23  13.8  .076 | 67  40.1  .863 | 51  30.5  .004 |  | 7  4.2  .000 | 9  5.4  .004 | 32  19.2  .421 |
| Arrest time > 30 min | 27  40.3  .029 | 38  56.7  .469 | 18  26.9  .937 | 15  22.4  .000 | 22  32.8  .464 | 16  23.9  .294 | 16  23.9  .000 | 13  19.4  .130 | 6  9.0  .593 | 10  14.9  .508 | 32  47.8  .191 | 25  37.3  .781 | 7  10.4  .000 |  | 2  3.0  .027 | 11  16.4  .304 |
| Admission > 10 days | 22  53.7  .869 | 21  51.2  .841 | 10  24.4  .749 | 4  9.8  .820 | 20  48.8  .091 | 9  22.0  .647 | 27  65.9  .002 | 7  17.1  .496 | 7  17.1  .171 | 7  17.1  .906 | 26  63.4  .002 | 25  61.0  .002 | 9  22.0  .004 | 2  4.9  .027 |  | 7  17.1  .506 |
| HLA mismatch > 4 | 47  57.3  .320 | 51  62.2  .053 | 25  30.5  .354 | 9  11.0  .953 | 30  36.6  .970 | 16  19.5  .952 | 43  52.4  .057 | 10  12.2  .671 | 9  11.0  .953 | 16  19.5  .636 | 33  40.2  .938 | 38  46.3  .116 | 32  39.0  .421 | 11  13.4  .304 | 7  8.5  .506 |  |

Data are N, %, p value. Positive correlation (p<0.05) darker shading, negative correlation (p<0.05) no shading

*Abbreviations for characteristics in the top row are expanded in the left column.

Supplementary Table S2. Outcome 12 months post-transplant in the development cohort.

|  |  |  | Post-transplant 12-month outcome (N=108) | | |
| --- | --- | --- | --- | --- | --- |
|  |  |  | OR | 95%CI | p value |
| Donor | |  |  |  |  |
| Age (years) | |  | 1.05 | 1.01 - 1.09 | .017 |
|  | Male gender |  | 0.59 | 0.24 - 1.44 | .245 |
|  | CrC (ml/min) continuous^a^ |  | 0.96 | 0.93 - 0.98 | .001 |
|  | < 70^b^ |  | 6.99 | 2.55 - 19.2 | .000 |
|  | Creatinine ≥ 100% rise |  | 1.10 | 0.21 - 5.83 | .909 |
|  | Urine < 75 ml/h |  | 3.47 | 1.37 - 8.78 | .009 |
|  | Proteinuria (> 1+) |  | 0.93 | 0.36 - 2.35 | .870 |
|  | Hypertension |  | 2.52 | 1.00 - 6.34 | .050 |
|  | Diabetes |  | 2.10 | 0.46 - 9.48 | .335 |
|  | Vascular event |  | 3.37 | 0.20 - 56.0 | .396 |
|  | Alcohol excess |  | 0.16 | 0.02 - 1.26 | .082 |
|  | Smoking |  | 0.47 | 0.17 - 1.30 | .145 |
|  | Donor cardiac death |  | 3.70 | 1.45 - 9.39 | .006 |
|  | Stroke (cause of death) |  | 0.43 | 0.16 - 1.14 | .089 |
|  | Arrest duration > 30 min |  | 1.21 | 0.46 - 3.18 | .703 |
|  | Admission ≥ 10 days |  | 3.59 | 0.68 - 19.0 | .133 |
|  | HLA mismatch ≥ 4 |  | 1.94 | 0.71 - 5.29 | .195 |
| Recipient (N=363) | |  |  |  |  |
| Age (years) | |  | 1.03 | 0.99 - 1.08 | .118 |
|  | Weight (kg) |  | 1.03 | 1.00 - 1.06 | .075 |
|  | CRF > 50% |  | 0.42 | 0.11 - 1.56 | .197 |
|  | Wait time (years) |  | 1.00 | 0.80 - 1.26 | .967 |

OR: odds ratio (univariate) for poor outcome (transplant failure or GFR<30), results shaded if p<0.10

^a^CrC: creatinine clearance, as continuous variable

^b^CrC: creatinine clearance, as threshold dependent on recipient weight: 60 (<65 kg), 70 (65-85 kg), 80 (>85 kg)

HLA: human leukocyte antigen

CRF: calculated reaction frequency

Capsule Sentence Summary

This study develops and validates ‘adjusted donor age’ as a score to guide kidney transplant decisions by assessing deceased donor organ quality, predicting post-transplant

outcomes, and improving transparency and shared decision-making between clinicians and patients in the UK and Germany.
